# Prognostic role of sentinel lymph node biopsy for patients with cutaneous melanoma: A retrospective study of surveillance, epidemiology, and end-result population-based data

**DOI:** 10.18632/oncotarget.10140

**Published:** 2016-06-17

**Authors:** Jie Chen, Yu Xu, Ye Zhou, Yanong Wang, Huiyan Zhu, Yingqiang Shi

**Affiliations:** ^1^ Department of Gastric Cancer and Soft Tissue Sarcomas, Fudan University Shanghai Cancer Center, and Department of Oncology, Shanghai Medical College, Fudan University, Shanghai, People's Republic of China

**Keywords:** melanoma, SLNB, SEER, survival analysis

## Abstract

Sentinel lymph node biopsy (SLNB) is a sensitive operation for finding micro-metastasis in patients with cutaneous melanoma without evidence of clinically positive lymph node findings. However, until now, no clinical trials or retrospective studies with large samples have been performed to investigate the clinical role of SLNB for cutaneous melanoma patients. In this study, we used the data of cutaneous melanoma from the Surveillance, Epidemiology, and End Results (SEER) database to compare overall survival (OS) and melanoma-specific survival (MSS) outcomes with clinical lymph node and SLN status. In total, 56,285 eligible patients were identified in this study. Cutaneous melanoma patients with clinically-positive lymph nodes had significantly shorter OS (46.1% vs 78.6%, *p* = 0.000) and MSS (55.8% vs 90.5, *p* = 0.000) compared with clinically-negative lymph node patients. Patients who underwent SLNB had significantly longer 5-year rates for OS (84.3% vs 70.1, *p* = 0.000) and MSS (91.5% vs 90.3, *p* = 0.000) compared with patients who did not undergo SLNB (lymph node observation). Patients with a negative SLNB had a significantly longer 5-year rate for OS (86.5% vs 68.1% vs 46.1, *p* = 0.000) and MSS (93.7% vs 75.1% 55.8%, *p* = 0.000) than patients who were SLNB-positive or had clinically-positive lymph nodes. This present study showed that the status of SLN is a valuable prognostic factor in patients with Breslow thickness greater than 1 mm in clinically-negative lymph node cutaneous melanoma.

## INTRODUCTION

Cutaneous melanoma is the fifteenth most common cancer in world, with more than 120,000 new cases diagnosed in 2015 [1]. It has long been recognized as a potentially aggressive form of skin cancer [2]. Despite many advances in the diagnosis, adjuvant therapy, and even targeted therapy of this disease, the prognosis for cutaneous melanoma remains poor. Several prognostic factors of OS and MSS with cutaneous melanoma have been identified in previous studies, such as tumor site, Breslow thickness, and ulceration, including sentinel lymph node biopsy (SLNB) [3, 4]. SLNB can detect micro-metastasis in the regional lymph node and is a powerful tool in the staging of cutaneous melanoma and is recommended in patients with thick Breslow depth. Until now, a few studies have proven the clinical value of SLNB; current treatment for cutaneous melanoma without metastasis is wide local excision and SLNB [5–7]. Moreover, SLNB for staging purposes is recommended by the American Joint Committee on Cancer (AJCC) Melanoma Staging Committee for all patients with primary tumors > 1 mm in Breslow thickness [8].

In this study, we aimed to evaluate the clinical usefulness of SLNB and to discuss the prognostic value of sentinel lymph node status for patients with cutaneous melanoma by using cutaneous melanoma data from the Surveillance, Epidemiology, and End Results (SEER) cancer-registry program of individuals diagnosed between 2004 and 2012.

## RESULTS

### Patient baseline characteristics

**Figure 1:**
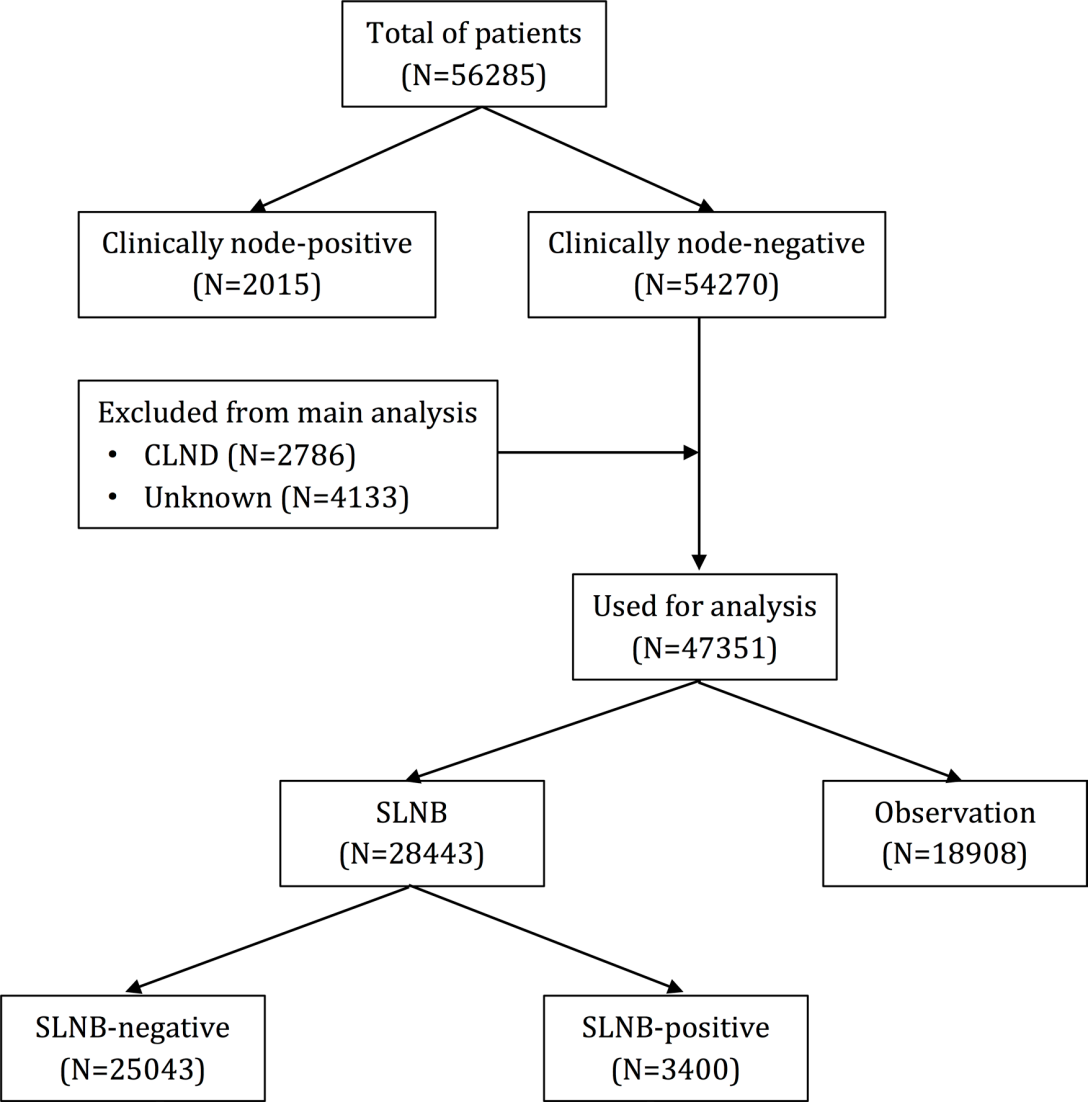
Flowchart of the study population

Between 2004 and 2012, a total of 56,285 eligible patients were identified with a new diagnosis of cutaneous melanoma with Breslow thickness greater than 1 mm during the 8-year period, including 34,466 male (61.2%) and 21,819 (38.8%) female patients. Median age was 63 years and median Breslow thickness was 1.50 mm. Lesions in 13,071 cutaneous melanoma patients were situated on the head and neck, 17,351 situated on the trunk, and 25,703 patients had lesions situated on extremities. In this study, nodular melanoma (NM) and superficial spreading melanoma (SSM) were the most common histotypes. Breslow thickness were between 1–2 mm in the majority of melanomas. The flowchart of the study population is shown in Figure 1 and the characteristics of cutaneous melanoma patients are listed in Table 1.

**Table 1:** Characteristics of 56285 patients with cutaneous melanoma

Characteristics	Total of patients (*N* = 56285)		Clinical node-negative (*N* = 47351)		SLNB (*N* = 28443)	
	Clinical node-positive (*N* = 2015)	Clinical node-negative (*N* = 54270)	Observation (*N* = 18908)	SLNB (*N* = 28443)	SLNB-positive (*N* = 3400)	SLNB-negative (*N* = 25043)
Gender						
male	1409	33057	11650	17012	2107	14905
female	606	21213	7258	11431	1293	10138
Age (y)						
≤ 60	990	23788	5907	14448	1897	12551
> 60	1025	30482	13001	13995	1503	12492
Primary site						
head/neck	424	12647	5996	4999	454	4545
trunk	697	16654	5328	9129	1323	7806
extremities	885	24818	7507	14268	1618	12650
unknown	9	151	77	47	5	42
Histotype						
Superficial spreading	316	14667	5027	8024	869	7155
nodular	659	8175	2434	4544	849	3695
desmoplastic	27	1411	525	718	24	694
lentiginous	88	2630	1191	1126	155	971
other/unknown	925	27387	9731	14031	1503	12528
Breslow depth (mm)						
1–2	502	35477	12714	17714	1206	16508
2–4	549	11434	3190	6811	1183	5628
≥ 4	964	7359	3004	3918	1011	2907
Ulceration						
yes	1175	13266	4152	7035	1476	5559
no	794	39354	14173	20579	1868	18711
unknown	46	1650	583	829	56	773

### SLN status

According to the flowchart of the study population, 2,015 patients (3.6%) presented with a positive clinical lymph node and underwent therapeutic lymph node dissection. 54,270 patients (96.4%) presented with clinically-negative lymph nodes and were excluded. Therapy information was unknown in patients who underwent completion lymph node dissection (CLND) despite having clinically negative SNB. Ultimately, 47,351 patients were used for analysis in this study. In these patients, SLNB was performed in 28,443 patients (60.1%), 3,400 patients presented with positive SLN and 25,043 patients presented with negative SLN (12% SLN positive rate). The remaining 18,908 patients underwent lymph node observation.

### Survival analysis

**Figure 2:**
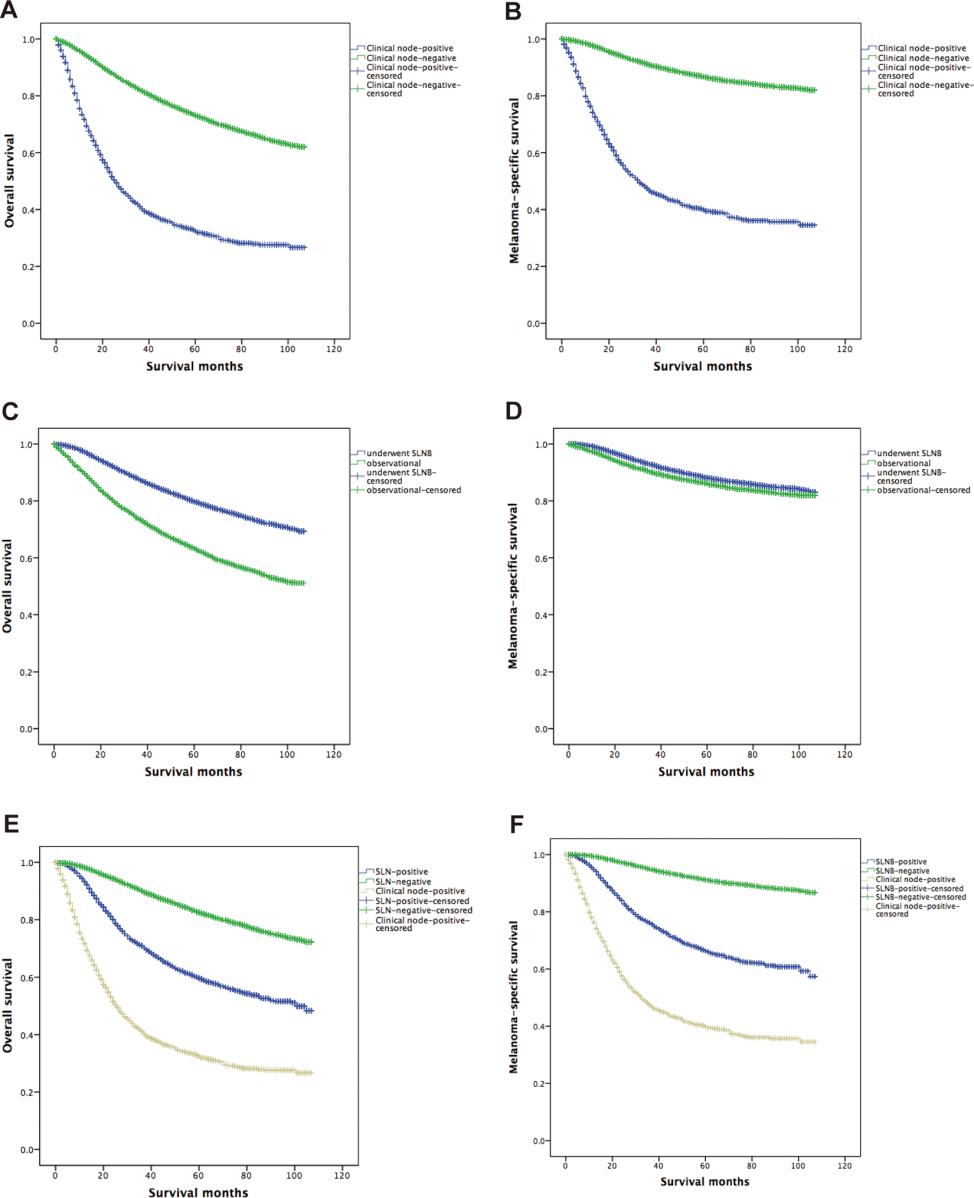
Kaplan-Meier curves are shown for overall survival and melanoma-specific survival Clinically positive indicates patients with clinically positive regional disease at the time of presentation who underwent therapeutic lymph node dissection; SLN, sentinel lymph node.

A total of 12,688 patients (24.1%) died during follow-up, and in these patients, 6,039 patients (47.6%) died of melanoma and 6,649 patients (52.4%) died of other diseases. Median follow-up time was 37.0 months. Patients who presented with clinically-positive lymph nodes had significantly shorter OS (46.1% vs 78.6%, *p* < 0.001, Figure 2A) and MSS (55.8% vs 90.5%, *p* < 0.001, Figure 2B) compared with patients who no clinical lymph nodes present. Patients who underwent SLNB had significantly longer 5-year survival rate for OS (84.3% vs 70.1%, *p* < 0.001, Figure 2C) and MSS (91.5% vs 90.3%, *p* < 0.001, Figure 2D) compared with patients who did not undergo SLNB (i.e., those who underwent lymph node observation). Patients with a negative SLNB had a significantly longer 5-year survival rate for OS (86.5% vs 68.1% vs 46.1%, *p* < 0.001, Figure 2E) and MSS (93.7% vs 75.1% vs 55.8%, *p* < 0.001, Figure 2F) than patients who were SLNB-positive and had clinical lymph node-positive disease.

### Univariate and multivariate analyses

Multivariate analysis showed that age (HR 1.896, 95% CI: 1.227–1.908, *P* < 0.001), primary site (HR 3.487, 95% CI: 2.125–4.009, *P* < 0.001), histotype (HR 1.843, 95% CI: 1.565-1.994, *P* < 0.001), Breslow depth (HR 1.453, 95% CI: 1.182-2.346, *P* < 0.001), ulceration (HR 2.367, 95% CI: 2.002–2.874, *P* < 0.001), status of clinical node (HR 1.236, 95% CI: 0.992–8.223, *P* < 0.001), and status of SLN (HR 1.083, 95% CI: 0.865–1.853, *P* < 0.001) were independent risk factors for OS. These factors were also independent prognostic factors associated with MSS. Tables 2 and 3 shows the risk factors related to OS and MSS.

**Table 2:** Univariate and multivariate analyses of factors associated with overall survival in patients with cutaneous melanoma

Factor	Univariate		Multivariate	
	HR (95% CI)	*P*	HR (95% CI)	*P*
Gender	1.675 (0.830–1.648)	0.134		NI
Age	1.541 (1.332–1.609)	< 0.001	1.896 (1.227–1.908)	< 0.001
Primary site	3.221 (1.088–4.632)	< 0.001	3.487 (2.125–4.009)	< 0.001
Histotype	1.062 (0.364–1.323)	< 0.001	1.843 (1.565–1.994)	< 0.001
Breslow depth	1.341 (1.118–2.165)	< 0.001	1.453 (1.182–2.346)	< 0.001
Ulceration	2.112 (1.988–2.427)	< 0.001	2.367 (2.002–2.874)	< 0.001
Status of clinical node	0.965 (0.898–4.756)	< 0.001	1.236 (0.992–8.223)	< 0.001
Status of SLN	1.043 (0.853–2.442)	< 0.001	1.562 (1.439–2.880)	< 0.001

NI: not included in the multivariate survival analysis.

**Table 3:** Univariate and multivariate analyses of factors associated with melanoma-specific survival in patients with cutaneous melanoma

Factor	Univariate		Multivariate	
	HR (95% CI)	*P*	HR (95% CI)	*P*
Gender	1.973 (1.335–2.876)	0.378		NI
Age	1.673 (1.556–1.923)	< 0.001	0.987 (0.665–1.632)	< 0.001
Primary site	3.887 (1.231–5.443)	< 0.001	1.689 (1.124–3.887)	< 0.001
Histotype	0.875 (0.586–1.545)	< 0.001	1.778 (1.345–2.334)	< 0.001
Breslow depth	1.122 (0.893–1.883)	< 0.001	1.086 (0.993–1.446)	< 0.001
Ulceration	1.776 (1.013–2.006)	< 0.001	2.157 (1.884–3.432)	< 0.001
Status of clinical node	0.778 (0.582–3.112)	< 0.001	1.187 (0.698–5.963)	< 0.001
Status of SLN	0.931 (0.845–1.975)	< 0.001	1.083 (0.865–1.853)	< 0.001

NI: not included in the multivariate survival analysis.

## DISCUSSION

SLN helps to predict the outcome of melanoma, and also allows clinicians to decide which patient may skip a completion lymphadenectomy and thus reduce morbidity. The status of the sentinel lymph node has been identified an important prognostic factor for cutaneous melanoma patients [9]. However, until now, there have not been large-sample clinical trials that have demonstrated this conclusion in overall survival or Melanoma-specific survival with the use of SLNB. Despite this, SLNB has become standard practice and has been recommended for clinically localized cutaneous melanoma with Breslow thickness between 1–4 mm and without metastasis [6, 10].

To our knowledge, a large prospective randomized trial named MSLT-I has evaluated cutaneous melanoma patients with clinically-negative lymph nodes who underwent SLNB while others were observed [3, 4]. The results showed that the prognostic significance of SLNB with five-year melanoma survival was 72% for SLN-positive patients compared to 90% survival for SLN-negative patients. Therefore, SLN status is a useful predictor of OS and MSS for patients with Breslow thickness between > 1 mm and without metastasis [10, 11].

According to previous studies, SLNB will detect nodal metastases in approximately 15–22% of cases in clinical lymph node-negative patients [12, 13]. These patients usually undergo completion lymphadenectomy and may be eligible for adjuvant therapy. The remaining 80–85% of patients with SLN-negative disease in general have a good prognosis: 5-year DFS is 88–90% and OS is 93% [14, 15]. SLNB has replaced radical regional lymph node dissection as a staging procedure with less morbidity. The SLN procedure is 95–98% accurate in staging the regional node basin and identifies the 15–20% of patients with nodal metastasis who require a complete lymph node dissection [16].

Until now, the status of sentinel lymph node helps to predict the outcome of melanoma. However, up to data, there is no large samples of clinical trials have demonstrated this conclusion in overall survival or Melanoma-specific survival with the use of SLNB. Therefore, the primary aim of this present study was to evaluate SLNB in patients with thick, clinically lymph node-negative melanoma to provide the relative value of SLNB by using large sample size of data from SEER database. The results showed that patients who performed SLNB had significantly longer OS and MSS compared with patients with did not undergo SLNB (lymph node observation). Patients with a negative SLNB had significantly longer OS and MSS than the patients who were SLNB-positive or clinically lymph node-positive.

This current study used the SEER database to investigate the value of SLNB and confirmed the prognostic role of SLNB for patients who were clinically lymph node-negative without metastasis. However, it still had several potential limitations. First, the SEER database lacks information on postoperative adjuvant therapy or target therapy in patients with cutaneous melanoma. Second, the survival outcomes of SLNB-positive patients with Breslow thickness > 4 mm is still unclear. Patients with thick melanoma have a higher risk of developing distant metastatic and guidelines suggested that SLNB “may be recommended”. Therefore, early control of regional disease may not impact survival [8].

In conclusion, this study shows that SLN status is a valuable prognostic factor in patients with Breslow thickness greater than 1 mm, clinically lymph node-negative cutaneous melanoma. Despite these potential limitations, SLNB is still recommended in patients with thick Breslow depth and without metastasis.

## MATERIALS AND METHODS

### Study population and data extracted

We used the SEER*Stat software to search for patients who were diagnosed with melanoma between 2004 and 2012 with a known SLNB status. The SEER Cancer Statistics Review (http://seer.cancer.gov/data/citation.html), a report on the most recent cancer incidence, mortality, survival, prevalence, and lifetime risk statistics, is published annually by the Data Analysis and Interpretation Branch of the National Cancer Institute, USA. The current SEER database consists of 17 population-based cancer registries that represent approximately 28% of the population of the United States. It contains no identifiers and is widely used for studies of the correlation between SLNB and OS or MSS information of patients with cutaneous melanoma.

In our study, we divided cutaneous melanoma patients into two groups according to clinical status of lymph nodes: (1) Clinically-positive nodes (2015); and (2) Clinically-negative nodes (54270). In clinically-negative node patients, they were further divided into two subgroups: (1) patients who underwent SLNB (28,443) and (2) observation group (18,908). Depending on the results of the SLNB, they were subsequently stratified into three groups: (1) SLNB-positive (3,400); (2) SLNB-negative (25,043); and (3) SLNB not performed (observation group, 18,908).

### Statistical analysis

The characteristics of cutaneous melanoma patients were extracted from the SEER database, including age, gender, tumor site, and Breslow thickness. Kaplan-Meier analyses were used to compare between groups by log-rank test. Survival outcomes of risk factors were analyzed by Multivariable Cox regression models for cutaneous melanoma patients. Statistical analyses were performed using the statistical software package SPSS for Windows, version 18.0 (SPSS Inc, Chicago, IL). All CIs were stated at the 95% confidence level. *P* values < 0.05 were considered statistically significant.
